# Systematic analysis of the *PTEN* 5′ leader identifies a major AUU initiated proteoform

**DOI:** 10.1098/rsob.150203

**Published:** 2016-05-25

**Authors:** Ioanna Tzani, Ivaylo P. Ivanov, Dmitri E. Andreev, Ruslan I. Dmitriev, Kellie A. Dean, Pavel V. Baranov, John F. Atkins, Gary Loughran

**Affiliations:** 1 School of Biochemistry and Cell Biology, University College Cork, Cork, Ireland; 2 Eunice Kennedy Shriver National Institute of Child Health and Human Development, National Institutes of Health, Bethesda, MD 20892, USA; 3 Belozersky Institute of Physico-Chemical Biology, Lomonosov Moscow State University, Moscow 119992, Russia; 4 Department of Human Genetics, University of Utah, Salt Lake City, UT 84112-5330, USA

**Keywords:** PTEN-L, non-AUG, AUU, uORF

## Abstract

Abundant evidence for translation within the 5′ leaders of many human genes is rapidly emerging, especially, because of the advent of ribosome profiling. In most cases, it is believed that the act of translation rather than the encoded peptide is important. However, the wealth of available sequencing data in recent years allows phylogenetic detection of sequences within 5′ leaders that have emerged under coding constraint and therefore allow for the prediction of functional 5′ leader translation. Using this approach, we previously predicted a CUG-initiated, 173 amino acid N-terminal extension to the human tumour suppressor PTEN. Here, a systematic experimental analysis of translation events in the *PTEN* 5′ leader identifies at least two additional non-AUG-initiated PTEN proteoforms that are expressed in most human cell lines tested. The most abundant extended PTEN proteoform initiates at a conserved AUU codon and extends the canonical AUG-initiated PTEN by 146 amino acids. All N-terminally extended PTEN proteoforms tested retain the ability to downregulate the PI3K pathway. We also provide evidence for the translation of two conserved AUG-initiated upstream open reading frames within the *PTEN* 5′ leader that control the ratio of PTEN proteoforms.

## 1. Introduction

The process of translation can be described in four steps: initiation, which is usually tightly regulated; elongation; termination; and ribosome recycling [1]. In eukaryotes, the scanning model for translation initiation postulates that the small ribosomal subunit, in complex with initiation factors and Met-tRNA_i_, binds first to the 5′ cap then scans 3′ until a suitable initiation codon is found [2]. Base-pairing interactions between the anticodon loop of the Met-tRNA_i_ bound to the ribosome and an AUG codon in the mRNA cause the ribosome to stop scanning and set the reading frame for protein synthesis [3]. Typically, the ribosome initiates protein synthesis at the AUG codon closest to the 5′ end of the mRNA, though the efficiency of initiation is dependent on the nucleotide sequence surrounding the initiator codon with the optimal sequence known as the Kozak context [4]. The Kozak context—comprising 6 nt before and 1 nt immediately following a potential initiation codon—has significant influence on the recognition of an initiation site, through partially understood mechanisms requiring the activities of eIF1 [5–8] and eIF5 [9–11]. Using multiple sequence alignments, the consensus context in mammals was identified as GCCRCC**AUG**G [12] with the identity of the underlined nucleotides in the −3 and +4 positions (relative to the ‘A’ of the AUG) being the most important. However, a recent high-throughput analysis of all possible initiation contexts revealed RYMRMVAUGGC as the optimal context in human and mouse cells and additionally revealed synergistic effects of neighbouring nucleotides [13].

Initiation can occur at most codons that differ from AUG by a single nucleotide (non-cognate or non-AUG). Seven out of the nine possible single-nucleotide substitutions at the AUG start codon of dihydrofolate reductase were functional as translation start sites in mammalian cells [14]. In all of the cases in which it was examined, the N-terminal residue of these proteins was methionine [14], suggesting that translation initiation relied on mis-pairing between the anticodon of Met-tRNA_i_ and the non-AUG start codon in the mRNA. However, a report exists of CUG initiation by elongator Leu-tRNA functioning as initiator tRNA [15].

Initiation is the only step where an incoming aminoacyl tRNA is bound directly in the ribosomal P-site [16,17]. Unlike the A-site, where mRNA : tRNA interactions are strictly monitored by the decoding centre [18], the P-site can tolerate mismatches in the codon : anticodon duplex [19–22]. This allows the incorporation of Met-tRNA_i_ at a wider range of codons compared with elongator Met-tRNA whose incorporation is strictly limited to AUG codons. The most favourable context for non-AUG initiation is believed to be identical to that for AUG starts [23–25]. In addition, a strong RNA secondary structure starting approximately 15 nt downstream of the non-AUG codon may significantly increase initiation efficiency [26]. Another important factor for non-AUG initiation is that it is located upstream of the most 5′ AUG codon [27].

When initiation codons occur in the 5′ leaders of transcripts they give rise to either N-terminal extensions to the main ORF or else upstream open reading frames (uORFs). It has been estimated that AUG-initiated uORFs are present in approximately half of the human protein coding genes [28]. Furthermore, ribosome profiling provided evidence for the presence of translating ribosomes on more than 200 non-AUG-initiated uORFs in yeast [29] and much more widespread non-AUG initiation in mammals [30–32]. In general, the translation of uORFs has an inhibitory effect on translation of the main protein coding ORF, because ribosomes terminating a uORF are often unable to reinitiate owing to the loss of necessary initiation factors. However, the 40S subunits of ribosomes translating short ORFs (less than 35 codons) may retain some initiation factors after termination [4,33]—although efficient re-initiation is precluded until all necessary initiation factors have been reloaded onto the 40S subunit. In most instances, there is a requirement for a ternary complex of eIF2, GTP and Met-tRNA_i_ which is regulated by the phosphorylation status of eIF2α [34].

An earlier finding that sequences in the 5′ leaders are highly conserved and that the level of conservation globally increases towards the leader/main ORF boundaries [35] suggests that this conservation could be due, in part, to the 3′ ends of a portion of 5′ leaders encoding N-terminal extensions to the annotated AUG-initiated proteins. In total, more than 60 instances of non-AUG-initiated N-terminal extensions have been predicted or verified experimentally in mammals [36–38]. In most cases, the non-AUG initiation provides an alternative longer proteoform in addition to a proteoform resulting from initiation at a standard AUG codon downstream via a process termed ‘leaky scanning’. Where alternative proteoforms are produced as a result of leaky scanning, the longer isoform frequently contains a signal for subcellular localization that is absent in the shorter form [39–43].

Previously, we performed a systematic analysis of the 5′ leaders of human GenBank RefSeq mRNAs to investigate the extent of non-AUG initiation in humans [37]. This involved analysis of codon substitution rates in pairwise alignments of human and mice orthologous sequences to identify regions of 5′ leaders evolving under the constraints of protein coding evolution. When a region within a 5′ leader evolves under such constraints, it is very likely that the encoded protein can improve an organism's fitness and is thus functional. This approach predicts a CUG-initiated, 173 amino acid, N-terminal extension within the 5′ leader of *phosphatase and tensin homologue on chromosome ten* (*PTEN*). *PTEN* is a powerful tumour suppressor gene that encodes a dual-specificity phosphatase [44,45] frequently mutated in human cancers [46] and autism spectrum disorders [47]. Its best characterized function is its ability to negatively regulate cell survival by dephosphorylating phosphatidylinositol 3,4,5 triphosphate (PIP3) and thus inhibiting phosphoinositide 3-kinase (PI3K) signalling [48].

Independently, two other groups subsequently identified the same PTEN N-terminal extension [49,50]. Here we extended our analysis of the *PTEN* 5′ leader and identify non-AUG-initiated translation that leads to the synthesis of at least two additional N-terminally extended proteoforms.

## 2. Material and methods

### 2.1. Plasmids

The *PTEN* 5′ leader was amplified by PCR from HEK-293T genomic DNA using appropriate primers (Integrated DNA Technologies) that incorporated a 5′ Hind*III* restriction site and a 3′ Bam*HI* restriction site. *PTEN* 5′ leaders were mutated by two-step PCR with appropriately designed primers. Amplicons were cloned Hind*III*/Bam*HI* into the dual luciferase plasmid p2-Luc [51] such that the *PTEN* 5′ leader replaced the *Renilla*-encoding sequences and were fused directly to the firefly-encoding sequences.

The coding sequence of PTEN was obtained as a gblock (Integrated DNA Technologies) with incorporated 5′ Hind*III* and a 3′ Xba*I* restriction site and cloned into phRL-CMV (Promega). The coding sequence of *PTEN* was also subcloned downstream of the *PTEN* 5′ leader to replace the firefly encoding sequence of the *PTEN* 5′ leader-FLuc constructs made previously.

For *PTEN* 5′ leader GFP fusions, EGFP was digested from pEGFP-N3 (Clontech) with Bam*HI* and Xba*I* restriction enzymes and cloned Bam*HI*/Xba*I* using standard cloning techniques into the *PTEN* 5′ leader firefly-encoding plasmids described above, such that the EGFP sequence replaced the firefly-encoding sequences and were fused directly to the *PTEN* 5′ leader. The EGFP AUG to AAA mutation was made by two-step PCR with appropriately designed primers.

For signal peptide–*Gaussia* luciferase fusions, amplicons generated by two-step PCR were cloned Bam*HI*/Xba*I* into pCMV-GLuc (NEB). All clones were verified by sequencing.

eIF1 and eIF5 overexpression constructs were described previously [52,53].

### 2.2. Cell culture

HEK-293T, MDA-MB-231, MCF-7, HeLa, HUH-7, U2OS and A172 cells were maintained in DMEM supplemented with 10% FBS, 1 mM l-glutamine and antibiotics. PC3 cells were maintained in RPMI supplemented with 10% FBS, 1 mM l-glutamine and antibiotics.

### 2.3. Luciferase assay

HEK-293T cells were transfected with Lipofectamine 2000 reagent (Invitrogen), using the 1 day protocol in which suspended cells are added directly to the DNA complexes in full-area 96-well plates. For each transfection, the following were added to each well: 100 ng of each firefly luciferase-expressing plasmid, 10 ng of each *Renilla* luciferase-expressing plasmid plus 0.4 µl Lipofectamine 2000 (Invitrogen) in 48.4 µl Opti-Mem (Gibco). The transfecting DNA complexes in each well were incubated with 4 × 10^4^ cells suspended in 50 µl DMEM (RPMI for PC3 cells) plus 10% FBS. Transfected cells were incubated at 37°C in 5% CO_2_ for 24 h. On the next day, cells were washed once with 1 × PBS and then lysed in 25 µl of 1 × passive lysis buffer (PLB; Promega) and firefly and *Renilla* luciferase activities were determined using the Dual Luciferase Stop & Glo^®^ Reporter Assay System (Promega). Relative light units were measured on a Veritas Microplate Luminometer with two injectors (Turner Biosystems).

Firefly luciferase activity was calculated relative to the activity of the co-transfected control plasmid expressing *Renilla* luciferase (pSV40-*Renilla*). All data points were averaged, and the standard deviation calculated. Data represent the mean and standard deviation of at least three independent experiments each done in quadruplicate.

For secretion luciferase assays (see figure 5*c*), only *Gaussia* luciferase activities were assayed and the percentage activity in both the cell lysate (intracellular) and culture media (extracellular) calculated. For sodium arsenite treatment, HEK-293T cells were transfected as above, and sodium arsenite (5 µM) was added 6 h post-transfection for either 2 or 4 h.

### 2.4. Immunoblotting

Cells were transfected in six-well plates using Lipofectamine 2000 reagent, again using the 1 day protocol described above, with 1 µg of each indicated plasmid. Where FLuc- and RLuc-expressing plasmids were cotransfected (see figure 6*b* and electronic supplementary material, figure S10*b*) a ratio of 10 : 1 was used. The transfecting DNA complexes in each well were incubated with 0.8 × 10^6^ HEK-293T cells suspended in 3 ml DMEM plus 10% FBS and incubated overnight at 37°C in 5% CO_2_. Transfected cells were lysed in 100 µl 1 × PLB and 10 µl removed for dual luciferase assay.

For PC3 transfections, 1.2 × 10^5^ cells were plated in triplicate wells (12-well plates) 1 day prior to forward transfection with Lipofectamine 2000 reagent (4 µl) and 500 ng of each indicated plasmid in 500 µl of Opti-Mem. Cells were replenished with fresh RPMI media 6 h post-transfection, then after 18 h, cells were washed and treated with serum free RPMI for a further 24 h. Transfected cells were lysed in radioimmunoprecipitation assay (RIPA) buffer plus protease inhibitors (Sigma) and NaF (20 mM).

Proteins were resolved by 4–12% gradient Bis/Tris–SDS/PAGE (Bolt^™^: Thermo Fisher Scientific) under constant voltage (165 V) for 90 min and transferred to nitrocellulose membranes (Protran), which were incubated at 4°C overnight with primary antibodies. Immunoreactive bands were detected on membranes after incubation with appropriate fluorescently labelled secondary antibody using a LI-COR Odyssey^®^ Infrared Imaging Scanner. Densitometry analysis was performed using ImageJ software (NIH) and GraphPad Prism used for statistical analysis.

### 2.5. Immunoprecipitation and GFP-trap^®^

Cells were lysed in RIPA buffer plus protease inhibitors (Sigma), then lysates were incubated with 25 µl of protein G agarose beads (Pierce) plus anti-PTEN (138G6) overnight at 4°C with gentle rocking. The beads were washed (three times) with ice-cold RIPA buffer and then immunoprecipitated proteins removed from the beads by boiling for 5 min. in 20 µl of 2 × SDS–PAGE sample buffer for electrophoresis and immunoblotting.

GFP-trap^®^_A beads (ChromoTek) were equilibrated according to the manufacturer's protocol. For collection of the extracellular fractions, culture medium was centrifuged at 200*g* for 5 min at 4°C to remove debris. For intracellular fractions, cells were lysed in RIPA buffer as above. 10 µl of equilibrated beads were added to each fraction and incubated rotating at 4°C for 1 h. The beads were washed (three times) with ice-cold dilution buffer (10 mM Tris/Cl pH 7.5; 150 mM NaCl; 0.5 mM EDTA) and then immunoprecipitated proteins removed from the beads by boiling for 10 min in 40 µl of 2 × SDS–PAGE sample buffer for electrophoresis and immunoblotting.

### 2.6. RT-qPCR

HEK-293T cells were transfected in triplicate wells (six-well plate) as above with either construct 1 (wild-type *PTEN* leader fused to firefly luciferase) or construct 5 (*PTEN* leader with all uAUGs mutated to AGG). 24 h post-transfection cells were removed (trypsin), divided into two aliquots of 20% and 80% and then collected by centrifugation. 20% of cells were resuspended in 20 µl 1 × PLB for dual luciferase assay. RNA was isolated using Trizol reagent (Invitrogen) from the remaining 80% of cells, and 500 ng of DNAse-treated (RQ1: Promega) RNA was reverse transcribed using oligo-dT and random hexamers according to the manufacturer's instructions (Superscritpt III: Invitrogen). Reactions minus reverse transcriptase were included to control for contaminating genomic or plasmid DNA. SYBR green (Qiagen) qPCR was performed on an Applied Biosystems 7300 real-time PCR system with firefly luciferase primers (sense TGGAGAGCAACTGCATAAGG and antisense ATTCCGCGTACGTGATGTT) and a set of intron-spanning control primers for GAPDH (sense AGCCTCCCGCTTCGCTCTCT and antisense CCAGGCGCCCAATACGACCA). Relative RNA quantitation was analysed using the Livak method (2^−ΔΔC^_T_) and used to normalize relative luciferase activities to relative RNA levels.

### 2.7. Antibodies

An affinity-purified rabbit polyclonal antibody (anti-PTEN-L) directed to a predicted antigen (PRHQQLLPSLSSFFFSHRLPD) within all four extended PTEN proteoforms was prepared by GenScript. The following commercially available antibodies were also used. Mouse anti-PTEN (6H2.1; Millipore), rabbit anti-PTEN (138G6: Cell Signalling), rabbit anti-GFP (A6455: Novex), goat anti-firefly luciferase (G7451: Promega), rabbit anti-S473-phospho-AKT (D9E: Cell Signalling), mouse anti-pan-AKT (40D4: Cell Signalling), mouse anti-*Renilla* luciferase (1D5.2 Millipore), rabbit anti-eIF5 (ab85913: Abcam) and mouse anti-β-actin (AC-15: Sigma). Anti-eIF1 was a generous gift from Ariel Stanhill (Technion-Israel Institute of Technology).

### 2.8. Fluorescence microscopy

Live cell imaging was performed as described before [54] using an inverted Axiovert 200 fluorescence microscope (Zeiss), equipped with 100×/1.4 Plan Apochromat oil-immersion objective (Zeiss), pulsed excitation module (470 nm, 590 nm LEDs), bandpass filters 510–560 nm and gated CCD camera (LaVision, Biotec). Briefly, HeLa cells were seeded onto eight-well chambers pre-coated with a mixture of collagen IV and poly-d-lysine (Ibidi), allowed to attach (24 h) and forward transfected for 24 h with plasmid DNAs encoding PTEN-leader–GFP fusions as indicated. Images were processed using ImSpector software (LaVision, Biotec) and combined in Adobe Illustrator CS2.

## 3. Results

**Figure 1.**
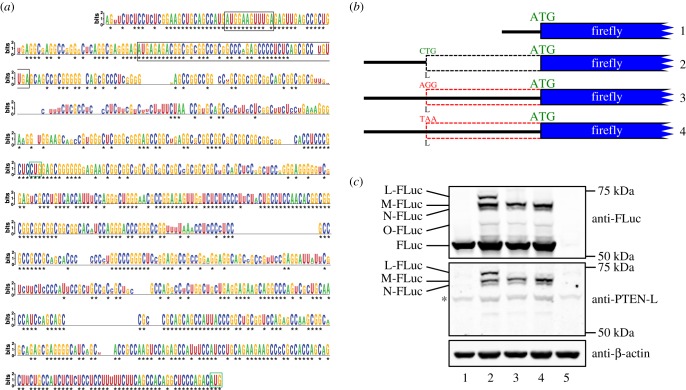
(*a*) Sequence logo representation (produced with WebLogo [55]) of a multiple sequence alignment of *PTEN* 5′ leaders from 52 mammals. The alignment was generated with ClustalX [56] and corrected manually. Asterisks indicate nucleotides conserved in all 52 species. Open black boxes represent two conserved uORFs. Open green boxes indicate the main ORF AUG and previously predicted CUG initiation codons. (*b*) Illustration of transfected firefly luciferase constructs 1–4 used for immunoblotting. (*c*) Immunoblot of cell lysates prepared from HEK-293T cells transfected with firefly luciferase expressing constructs as indicated and probed with antibodies against firefly luciferase (anti-FLuc: top panel), the PTEN N-terminal extension (anti-PTEN-L: middle panel) and β-actin (bottom panel). The four proteoforms with extended PTEN N-termini are named as L, M, N and O. This nomenclature was recently proposed for novel PTEN proteoforms by Pulido *et al.* [57]. Asterisk indicates a non-specific protein that co-migrates with the O-proteoform, thus precluding its detection with anti-PTEN-L. Lane 5 contains cell lysates from mock-transfected cells.

Previous searches for evolutionarily conserved non-AUG-initiated N-terminal extensions in human coding sequences predicted a CUG-initiated, 173 amino acid extension to the tumour suppressor PTEN [37]. Further phylogenetic analysis of the *PTEN* 5′ leader with additional sequence data reveals deep nucleotide conservation in mammals (figure 1*a* and electronic supplementary material, figure S1). Two independent groups [49,50] have recently provided experimental evidence for a human PTEN N-terminal extension that reportedly initiates at the same CUG predicted by Ivanov *et al.* [37]. Preliminary results in our laboratory indicated the existence of multiple N-terminally extended PTEN proteoforms. Here we set out to systematically investigate these multiple proteoforms and also to determine the effect, if any, of two conserved AUG-initiated uORFs on translation of these PTEN proteoforms (boxed in figure 1*a*).

We noted that the 5′ end of both GenBank RefSeq *PTEN* mRNA isoforms (NM_000314.6, NM_001304718.1), which have identical first exons, do not correspond to the transcription start site predicted by the Fantom Projects' CAGE analysis [58] which finds that the transcription start site is a further 187 nt 3′ of the Genbank RefSeq annotated *PTEN* mRNA 5′ end (electronic supplementary material, figure S2). A +187 transcription start site is also in agreement with mRNAseq data obtained as controls to multiple ribosome profiling experiments, available in GWIPS-viz [59] (see electronic supplementary material, figure S2) as well as with the majority of publicly available human *PTEN* expressed sequence tags. Therefore, in this study, all test constructs with the *PTEN* 5′ leader start at +187 relative to the 5′ end of GenBank RefSeq *PTEN* mRNAs.

We first transfected HEK-293T cells with plasmid DNA expressing the human *PTEN* 5′ leader fused to sequences encoding firefly luciferase (FLuc; figure 1*b*). Immunoblots generated from transfected cell lysates and probed with FLuc antibodies detected four slower migrating FLuc proteoforms when FLuc is preceded by the *PTEN* 5′ leader (lane 2, top panel, figure 1*c*). These four proteoforms are absent from cells transfected with FLuc expressing constructs lacking the *PTEN* 5′ leader (lane 1, top panel, figure 1*c*) indicating that the multiple proteoforms are not post-translationally modified variants of FLuc. Furthermore, the same pattern of proteoforms was also detected when these same lysates were probed with a custom antibody (anti-PTEN-L) directed against a peptide predicted from sequences immediately 5′ of, and in-frame with, the *PTEN* main ORF (lane 2, middle panel, figure 1*c*).

These multiple proteoforms are most likely N-terminally extended variants generated by initiation at in-frame non-AUG codons within the *PTEN* 5′ leader. Alternatively, some of these proteoforms could be proteolytically cleaved variants of the previously reported [49,50] CUG-initiated 173 amino acid N-terminally extended PTEN proteoform. To investigate this latter possibility, we transfected HEK-293T cells with *PTEN* 5′ leader-FLuc expressing constructs in which this CUG was changed to either a non-initiating AGG triplet or to a UAA stop codon, that not only prevents initiation but also terminates translation from upstream initiation sites. Both anti-FLuc and anti-PTEN-L immunoblots from these transfected lysates indicate that mutation of the CUG leads to the disappearance of only the most slowly migrating proteoform, thus ruling out the possibility that some of the other proteoforms are cleavage products (lanes 3 and 4, top and middle panels, figure 1*c*). These observations suggest that the *PTEN* 5′ leader has the potential to generate at least four N-terminally extended proteoforms (that are within the detection limits of these experiments) and that the previously reported proteoform initiated at CUG, while likely the longest proteoform may not be the most abundant.

Because of the recent reporting of an N-terminally extended PTEN proteoform [37,49,50], a unified nomenclature for PTEN proteoforms was proposed [57] where newly identified PTEN proteins are named alphabetically as PTEN-L, PTEN-M, PTEN-N, etc. We have adopted this proposed nomenclature and henceforth refer to these four PTEN isoforms as PTEN-L, PTEN-M, PTEN-N and PTEN-O, where PTEN-L is the presumed longest variant and is initiated at the previously reported CUG codon [37,49,50], whereas proteoform PTEN-M appears to be the most abundant (table 1 and figure 1*c*).

**Table 1.** Details of PTEN N-terminally extended proteoforms. Nucleotide distances from aAUG (annotated AUG of canonical PTEN) are indicated where A of the aAUG is +1.

	nt from aAUG	N-term ext AA	total nt	total AA	MW (kDa)
PTEN-L	−519	173	1728	576	64.9
PTEN-M	−438	146	1647	549	62.5
PTEN-N	−393	131	1602	534	61.0
PTEN-O	−216	72	1425	475	55.0

**Figure 2.**
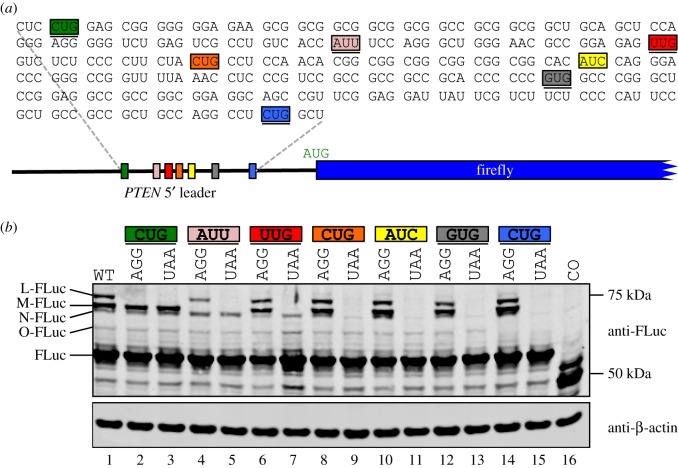
(*a*) Illustration of human *PTEN* 5′ leader fused to firefly luciferase and sequence of 5′ leader region in which potential non-AUG codons are highlighted in different colours with those in favourable Kozak context (−3 purine and/or +4 G) underlined. The previously reported CUG initiation site [37,49,50] is highlighted in green and is the most 5′ potential non-AUG initiation site. The *PTEN* CDS reading frame is indicated with spaces between the codons. (*b*) Anti-FLuc and anti-β-actin immunoblots of cell lysates prepared from HEK-293T cells transfected with firefly luciferase encoding sequences fused to the wild-type or non-AUG codon mutated (indicated) *PTEN* 5′ leader. The four FLuc proteoforms with extended PTEN N-termini are indicated as L-, M-, N- and O-FLuc. In the control (CO, lane 16), the main ORF (firefly) AUG is mutated to UAA.

It is conceivable that PTEN-M, PTEN-N and PTEN-O are not non-AUG-initiated N-terminally extended proteoforms. Instead, PTEN-M and PTEN-N could be post-translationally modified variants of PTEN-O, whereas PTEN-N and PTEN-O could be cleavage products of PTEN-M. To address these possibilities, and to determine whether non-AUG initiation could explain the presence of these proteoforms, we made *PTEN* 5′ leader-FLuc constructs in which potential non-AUG initiation codons were systematically mutated in turn to either AGG or UAA. Potential in-frame near-cognate initiation codons from the relevant region within the *PTEN* 5′ leader are highlighted in figure 2*a* and those in a favourable Kozak context (purine at −3 or G at +4) are underlined. Mutation of the most 5′ in-frame AUU codon to AGG completely abolishes synthesis of the most prevalent proteoform M-FLuc (lanes 4 and 5, figure 2*b*). As expected, mutation of this AUU to a termination codon causes premature termination of L-FLuc (lane 5, figure 2*b*). It is difficult to be certain about the nature of the initiation codon for N-FLuc, because a cross-reacting endogenous protein migrates at the same position; however, it seems likely that the second-most 5′ in-frame CUG is responsible as there is a clear decrease in N-FLuc intensity when this CUG and all 3′ non-AUGs are mutated individually to UAA (see lanes 9, 11, 13 and 15, figure 2*b*). Mutation of the most 3′ in-frame CUG completely abrogates expression of O-FLuc (lanes 14 and 15, figure 2*b*). In summary, the three minor proteoforms (L, N and O) are all initiated at CUG, whereas the major proteoform (M) is initiated at AUU.

**Figure 3.**
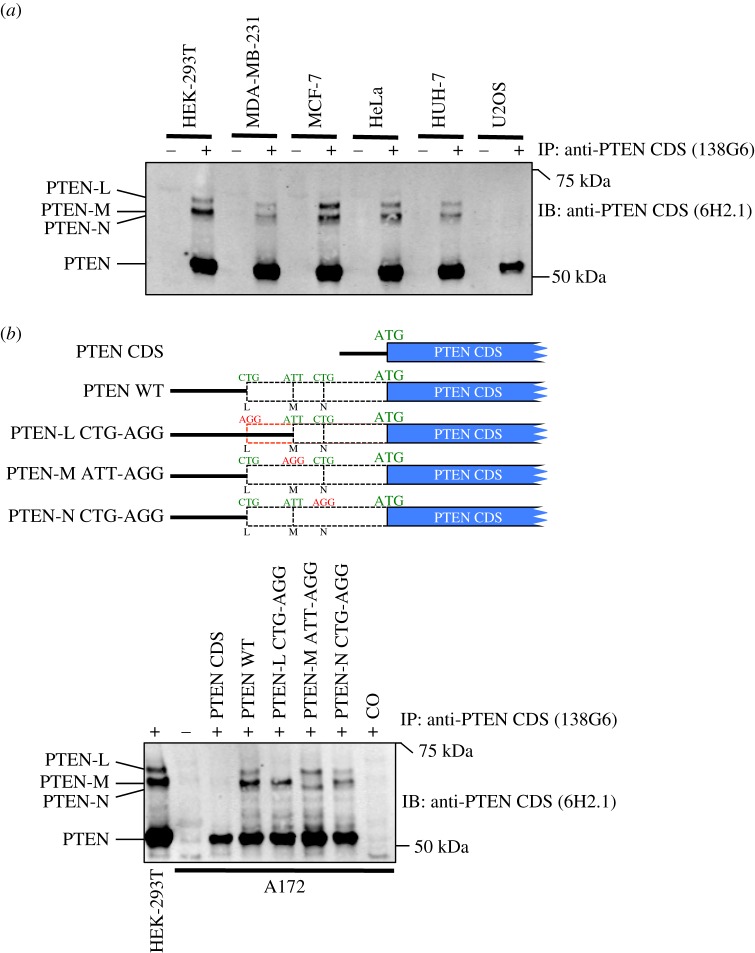
(*a*) Anti-PTEN CDS (6H2.1) immunoblot of anti-PTEN CDS (138G6) immunoprecipitates prepared from several cell lines as indicated showing detection of endogenous canonical AUG-initiated PTEN as well as the three non-AUG-initiated PTEN proteoforms PTEN-L, PTEN-M and PTEN-N. (*b*) Anti-PTEN CDS (6H2.1) immunoblot of anti-PTEN CDS (138G6) immunoprecipitates prepared from A172 cells (which lack endogenous PTEN) transfected with constructs expressing either wild-type PTEN or mutants of PTEN proteoforms PTEN-L, PTEN-M and PTEN-N (top panel illustration) showing that the non-AUG initiation codons of PTEN-L, PTEN-M and PTEN-N are CUG, AUU and CUG, respectively. The left-most lane shows control immunoprecipitates from HEK-293T cells indicating endogenous canonical AUG-initiated PTEN and N-terminally extended proteoforms PTEN-L, PTEN-M and PTEN-N. CO indicates control immunoprecipitates from mock-transfected cells.

We next tested for the existence of endogenous human PTEN N-terminally extended proteoforms by immunoprecipitating PTEN from several different cell lines using commercially available antibodies directed against antigens within the annotated PTEN (CDS). The predicted molecular weight of PTEN is 47.2 kDa, but there are many reports indicating that the apparent molecular weight of PTEN is approximately 55 kDa. In agreement with this, immunoblots of PTEN immunocomplexes reveal the presence of an approximately 55 kDa protein in all cell lines tested (figure 3*a*). In addition to the canonical AUG-initiated PTEN, three slower migrating proteins are observed in all cell lines tested other than U2OS. The molecular weights of these three proteins correlate well with those predicted for PTEN proteoforms PTEN-L, PTEN-M and PTEN-N. To determine whether the migration of these putative endogenously expressed PTEN N-terminally extended proteoforms correlate with exogenously expressed proteoforms PTEN-L, PTEN-M and PTEN-N (we could not detect any endogenous proteoform that could correspond in molecular weight to PTEN-O in these experiments), we transfected the human *PTEN* CDS fused to either wild-type or mutated *PTEN* 5′ leaders into A172 cells which do not express endogenous *PTEN*. Immunoprecipitates from transfected A172 cells were compared with immunoprecipitates from HEK-293T cells expressing endogenous *PTEN* and show that exogenously expressed proteoforms PTEN-L, PTEN-M and PTEN-N co-migrate with endogenous PTEN proteins from HEK-293T cells (compare the first and fourth lanes in figure 3*b*). Furthermore, in agreement with the FLuc reporter constructs (figure 2*b*), mutation of the first and third in-frame CUGs prevents initiation of the two minor PTEN proteoforms PTEN-L and PTEN-N, whereas mutation of the first in-frame AUU abolishes expression of the major PTEN-M proteoform (figure 3*b*). Together, these results indicate that three slower migrating PTEN proteoforms apparent in immunoprecipitates from several cell lines correlate with non-AUG-initiated proteoforms PTEN-L, PTEN-M and PTEN-N.

**Figure 4.**
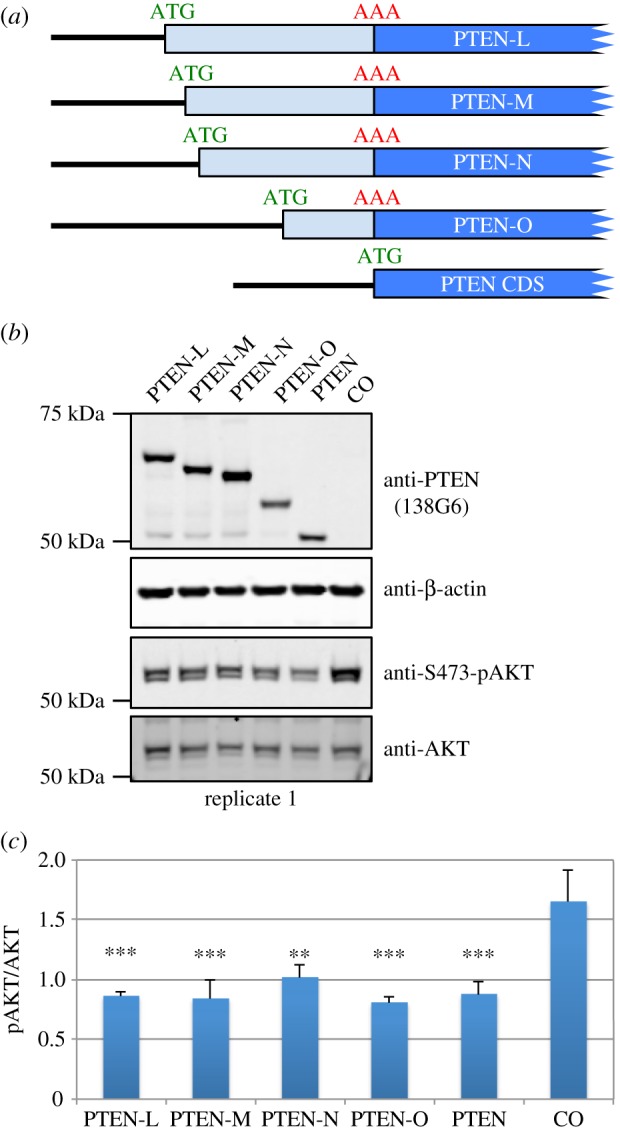
(*a*) Illustration shows PTEN expressing constructs transfected into PC3 cells. (*b*) Immunoblots of cell lysates prepared from PTEN-null PC3 cells transfected with PTEN expressing constructs as indicated for 48 h (serum starved for last 24 h) and probed with antibodies against PTEN (138G6), β-actin, phospho-AKT (S473) and pan-AKT. Additional replicates (replicates 2 and 3) are shown in the electronic supplementary material, figure S3. (*c*) Mean and standard deviations of relative protein intensities determined by densitometry from three biological replicates. Phospho-AKT intensities were calculated relative to pan-AKT intensities. Relative pAKT levels in lysates from cells transfected with each N-terminally extended PTEN proteoform were compared with the control sample. **p < 0.01, ***p < 0.001 by one-way ANOVA followed by Tukey's test.

Several studies report that PTEN-L is an active phosphatase and retains the ability to downregulate the PI3K pathway [49,50,60–62]. To determine whether the PTEN proteoforms described here are active phosphatases, we measured the phosphorylation status of the major PI3K substrate, AKT, in PC3 cells (no endogenous PTEN expression) transfected with either wild-type or N-terminally extended PTEN proteoforms. Exogenous expression of PTEN reduced the levels of AKT phosphorylation almost twofold, and similar levels of reduction were observed for all four N-terminally extended proteoforms (figure 4 and electronic supplementary material, figure S3). Therefore, similar to previous observations for PTEN-L, the phosphatase activities of PTEN-M, PTEN-N and PTEN-O are not overtly affected by their N-terminal extensions.

N-terminal extensions often harbour signals for subcellular targeting or secretion. However, live cell imaging of AUG-initiated PTEN N-terminal extensions fused to GFP reveal diffuse cytoplasmic localization for all four PTEN–GFP fusions indistinguishable from the localization of GFP alone (electronic supplementary material, figure S4).

**Figure 5.**
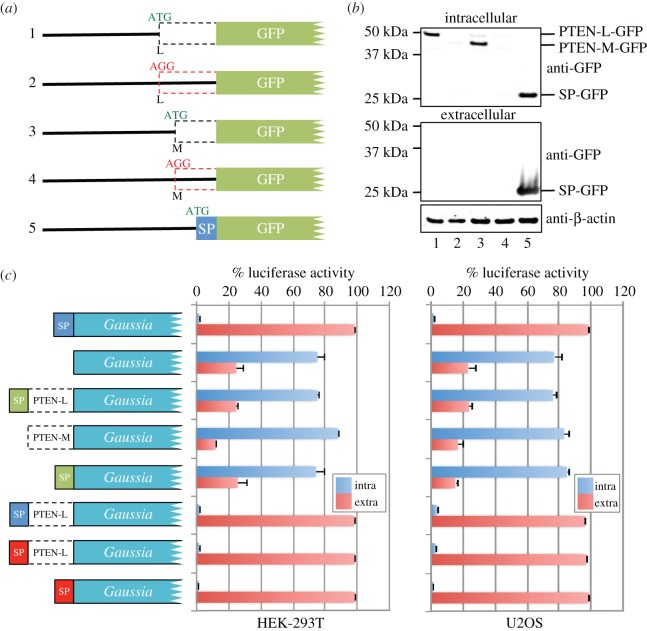
(*a*) Illustration of mutant *PTEN-L* and *PTEN-M* 5′ leader–GFP fusion constructs transfected into HEK-293T cells. SP is the secretion peptide from *Gaussia* luciferase. (*b*) Anti-GFP and anti-β-actin immunoblots from GFP-trap immunoprecipitates prepared from either HEK-293T cell lysates (intracellular: top panel) or culture media (extracellular: middle panel) transfected with the GFP fusion constructs shown in (*a*). (*c*) *Gaussia* luciferase assays show the percentage luciferase activity in the cell lysate (blue: intracellular) and culture media (red: extracellular) when either HEK-293T or U2OS cells (as indicated) were transfected with the constructs indicated for 22 h. The blue signal peptide (SP) is from *Gaussia* luciferase (MGVKVLFALICIAVAEAK), the putative PTEN-L SP (MERGGEAAAAAAAAAAAPGRG) is in green and the interleukin-2 (IL2) SP (MYRMQLLSCIALSLALVTNSA) is in red.

Hopkins *et al.* [49] suggest that the PTEN-L proteoform harbours an N-terminal signal peptide secretion signal and provide evidence that PTEN-L is secreted and can re-enter cells via a cell re-entry motif similar to the HIV TAT protein. The predicted amino acid sequences of PTEN-M and PTEN-N lack the putative secretion signal yet still retain the putative cell re-entry motif (electronic supplementary material, figure S5) reported by Hopkins *et al*. To rule out the possibility that the N-terminal extension of PTEN-M might possess a cryptic signal peptide sequence, we overexpressed, in HEK-293T cells, PTEN-L (as a control) and PTEN-M N-terminal extensions fused to GFP (same constructs as described in electronic supplementary material, figure S4). A fusion of GFP with the signal peptide sequence from *Gaussia* luciferase (GLuc) [63] was used as a positive control (figure 5*a*). Extracellular and intracellular GFP fusion proteins were concentrated by immunoprecipitation using GFP-trap^®^ (immobilized camelid anti-GFP antibody) followed by immunoblotting with anti-GFP. Even though we immunoblotted 50% of the total extracellular fraction and only 5% of the intracellular fraction, we did not detect PTEN-L-GFP or PTEN-M-GFP in the extracellular fraction (see lanes 1 and 3, middle panel, figure 5*b*). Furthermore, when HEK-293T or U2OS cells were transfected with constructs expressing either the PTEN-L or PTEN-M N-terminal extensions, or the putative PTEN-L signal peptide alone, fused to GLuc, we failed to detect GLuc activity in the cell media at levels above background (figure 5*c*). Yet GLuc-fused downstream of the PTEN-L N-terminal extension in which the putative PTEN-L signal peptide was replaced with the signal peptide from either GLuc or interleukin-2 efficiently targeted GLuc from cells (figure 5*c*).

We next sought to ascertain whether the non-AUG initiation of the PTEN proteoforms is regulated. The selection of poor context initiation codons (including non-AUG start codons) is modulated by intracellular levels of initiation factors eIF1 (increases stringency) and eIF5 (decreases stringency) [5–11,52,53]. To determine whether initiation of the N-terminally extended PTEN proteoforms are regulated by altered eIF1 or eIF5 levels, we overexpressed each initiation factor separately in HEK-293T cells and then immunoprecipitated endogenous PTEN proteoforms (electronic supplementary material, figure S6). Even though eIF1 and eIF5 levels are robustly expressed, we note no discernible change in the ratio of PTEN proteoforms compared with cells transfected with an empty vector (electronic supplementary material, figure S6).

**Figure 6.**
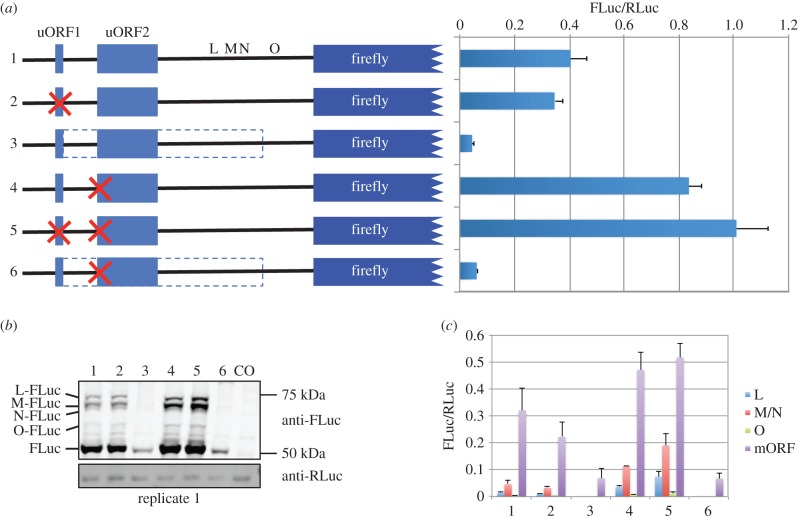
(*a*) Relative luciferase activities (FLuc/RLuc) of firefly encoding sequences fused to the wild-type or mutant *PTEN* 5′ leader as indicated and cotransfected (10 : 1 ratio) with a *Renilla* expressing plasmid into HEK-293T cells. Red crosses indicate mutation of AUG start codons to non-initiating AGG codons. L, M, N and O depict the approximate site of initiation of PTEN extensions. The dashed box represents the increase in ORF length when the stop codon of uORF1 is changed to a sense codon. (*b*) Anti-FLuc and anti-RLuc immunoblots of cell lysates prepared from HEK-293T cells transfected with *Renilla* and firefly luciferase expressing constructs indicated in (*a*). The four proteoforms with extended PTEN N-termini are indicated as L, M, N and O. CO represents lysates prepared from mock-transfected cells. (*c*) Densitometry analysis from three biological replicates of the proteins detected by anti-FLuc and anti-RLuc in (*b*) and electronic supplementary material, figure S10*b*. Proteoforms M and N could not be resolved sufficiently from each other for accurate densitometry analysis, so the intensity of both proteins together is determined. FLuc intensities were calculated relative to RLuc.

As shown in figure 1*a*, there are two conserved AUG-initiated uORFs (uORF1 and uORF2) close to the *PTEN* 5′ cap (figure 6*a* and electronic supplementary material, figure S7). The most 5′ uORF (uORF1) is only four codons long and starts with tandem AUG codons, both in good Kozak context. uORF2 is much longer (45 codons) and also starts with a good context AUG. Ribosomal profiling data compiled in GWIPS-viz [59] for *PTEN* show a large number of ribosome protected fragments aligning to uORF1 in comparison with uORF2 (electronic supplementary material, figure S7). In general, translation of uORFs has an inhibitory effect on main ORF translation although this relationship between uORFs and main ORFs is not so simple where multiple uORFs exist. Usually, the translation of short uORFs is less inhibitory than the translation of long uORFs, because the ribosomes' ability to reinitiate after translation of ORFs more than 35 codons is normally greatly reduced (see Introduction). According to the scanning model of eukaryotic translation initiation, we would predict that the majority of scanning 43S complexes would initiate uORF1; however, because it is only four codons long, a high level of re-initiation is expected. Because re-initiation is precluded until all necessary initiation factors have reloaded onto the 40S, translation of uORF1 may favour re-initiation at ORFs more 3′ than uORF2. We set out to determine the possible role of these two uORFs on regulation of the different PTEN proteoforms by transfecting HEK-293T cells with *PTEN* 5′-leader-FLuc reporter constructs in which the uORFs were mutated and testing by dual luciferase assay (figure 6*a*) and immunoblotting (figure 6*b*).

Even though we expect a high level of uORF1 translation, mutation of the uORF1 tandem AUG codons to non-initiating AGG codons has only a minor inhibitory effect (less than 10%) on FLuc activity (compare construct 1 (wild-type *PTEN* 5′ leader) with construct 2 in figure 6*a*). Mutating uORF1 is inhibitory rather than stimulatory presumably because more ribosomes now have access to uORF2 which we predict should be inhibitory. However, the fact that the inhibition is only minor suggests that either uORF2 may not be as inhibitory as we expect or that normally ribosomes translating uORF1 can efficiently reinitiate uORF2. Another explanation could be that in the wild-type context many ribosomes do not initiate uORF1. However, mutation of the uORF1 stop codon to a sense codon (extending the length of uORF1 to 161 codons) severely diminishes FLuc activity (construct 3, figure 6*a*) which, in agreement with published ribosome profiling data (electronic supplementary material, figure S7), confirms that ribosomes do initiate at uORF1. A mutation that disables uORF2 translation, either alone or in combination with mutations that prevent uORF1 initiation, increased FLuc activity more than twofold (compare construct 1 (wild-type) with constructs 4 and 5 in figure 6*a*). This indicates that uORF2 is inhibitory and thus suggests that the level of re-initiation on uORF2 is high. Similar results were observed in several other cell lines tested, including breast carcinoma (MCF-7), prostate carcinoma (PC3) and cervical carcinoma (HeLa; electronic supplementary material, figure S8). In addition, there is little difference in steady-state mRNA levels when both uORFs are disabled (electronic supplementary material, figure S9).

In these reporter assays, it is assumed that the low level of N-terminally extended proteoforms (relative to the main ORF) have only a minor contribution to total FLuc activity. This is supported by our own unpublished observations showing that an AUG-initiated PTEN-L extension severely reduces FLuc activity. To gain further understanding of the effect of the uORFs on downstream translation, we performed anti-FLuc immunoblots from cells transfected with constructs 1–6 (figure 6*b* and electronic supplementary material, figure S10). Dual luciferase assays from replicate lysates show similar FLuc activity for each construct to those shown in figure 6*a* and electronic supplementary material, figure S8. Densitometry of the main ORF (FLuc) normalized to cotransfected RLuc levels indicates that, similar to the luciferase assay results, preventing initiation of the uORFs results in an almost twofold increase in main ORF levels (figure 6*c*). Therefore, even though levels of the N-terminally extended proteoforms increase when uORF2 is mutated (lanes 4 and 5, figure 6*b* and electronic supplementary material, figure S10), they have only a minor contribution to overall FLuc activity. When we estimate the levels of each N-terminally extended proteoform by densitometry, we observe that mutation of uORF2 causes the levels of the L and M/N proteoforms to increase approximately 2.5-fold, the O proteoform to increase 2-fold, whereas the main ORF increases approximately 1.5-fold. Mutation of both uORFs together increases the L-proteoform approximately 5.3-fold, M/N-proteoforms approximately 4.1-fold, O-proteoform approximately 3.1-fold and the main ORF proteoform approximately 1.6-fold. This densitometry analysis also allows us to estimate the relative abundance of each proteoform under normal conditions and when ribosomes do not translate either or both uORFs. Interestingly, although all proteoforms increase when ribosomes do not translate uORF2, the ratio of N-terminally extended proteoforms relative to each other does not change (electronic supplementary material, figure S10*c*). In contrast, mutation of uORF2 increases the ratio of N-terminal proteoforms relative to the main ORF proteoform (electronic supplementary material, figure S10*d*) such that proteoforms M/N increase by 50% from 12% to 18% of all proteoforms. Similar approximately 50% increases were also observed for the L- and O-proteoforms (electronic supplementary material, figure S10*d*). Furthermore, there is a concomitant decrease in the relative abundance of the main ORF proteoform from 83% down to 75%. Mutation of uORF1 and uORF2 together results in even further increases to the relative abundance of N-terminally extended proteoforms and further decreases the main ORF proteoform (electronic supplementary material, figure S10*d*).

Because we show that translation of uORF2 can alter the ratio of extended and main ORF proteoforms and uORF2 translation seems to be dependent on efficient re-initiation after translation of uORF1, we predict that conditions which can regulate re-initiation events could alter translation of the main ORF. Increasing the phosphorylation status of eIF2 can reduce re-initiation by limiting the pool of functional (non-phosphorylated) eIF2. To gain some understanding of possible regulation of these uORFs, we transfected wild-type and ‘no uAUG’ firefly reporters into HEK-293T cells and then treated with sodium arsenite. Sodium arsenite activates the integrated stress response by inducing phosphorylation of eIF2. Many mRNAs (main ORFs) whose translation is resistant to eIF2 phosphorylation harbour translated uORFs [64]. If sodium arsenite decreased re-initiation on uORF2, then we would expect to see an increase in FLuc activity with the wild-type reporter; however, we observed little difference in main ORF reporter translation upon sodium arsenite treatment (electronic supplementary material, figure S11).

## 4. Discussion

The results presented above provide strong evidence for the existence of three (or perhaps four) non-AUG-initiated proteoforms of PTEN that are expressed in human cells in addition to, and at lower levels than, the well-studied canonical AUG-initiated PTEN. The longest PTEN proteoform, PTEN-L, has previously been reported [37,49,50] and its function has been investigated in more detail in subsequent studies [60,61,65–67]. However, PTEN-M, PTEN-N and PTEN-O have not been previously described and are reported here for the first time. We show that PTEN-M initiates at an AUU codon that is completely conserved in 52 eutherian mammalian species with available sequences, whereas the other PTEN proteoforms (PTEN-L, PTEN-N and PTEN-O) initiate at CUG, the first two also completely conserved while the latter only partially conserved. CUG codons are generally better initiators than AUU codons [14,52,53], so it is somewhat surprising that AUU-initiated PTEN-M is more abundant than CUG-initiated PTEN-L, especially, because the CUG is more 5′ than the AUU. The PTEN-L CUG initiation codon Kozak context is slightly less favourable (C at −3 and G at +4) than that of the PTEN-M AUU initiation codon (A at −3). It is also possible that an as yet unidentified RNA secondary structure 3′ of the AUU codon contributes to its favourable utilization for initiation (see Introduction), especially because the *PTEN* 5′ leader is 70% GC rich with numerous potential stem-loops. Yet another possibility is that the abundance of the PTEN proteoforms is a reflection of differing protein stabilities rather than initiation levels, although a similar level of exogenous PTEN-L and PTEN-M expression when their non-AUGs start codons are mutated to AUGs (figure 4*b* and electronic supplementary material, figure S3) would argue against this.

The identity of the major non-AUG-initiated PTEN proteoforms identified in this study contrasts with the findings of both Hopkins *et al.* [49] and Liang *et al.* [50], who both report only a single CUG-initiated proteoform that corresponds in our study to PTEN-L. Most of the anti-PTEN immunoblots presented by Hopkins *et al.* show a single slower migrating approximately 75 kDa PTEN proteoform which they term PTEN-Long (PTEN-L in our study). A possible reason for this discrepancy is that, in their study, proteins were separated for shorter time intervals while we purposely allowed SDS–PAGE gels to run for extended periods (see Material and methods) in an attempt to resolve as many PTEN proteoforms as technically possible. Perhaps the approximately 75 kDa PTEN-long detected by Hopkins *et al.* corresponds to a mixture of unresolved PTEN-L, PTEN-M and PTEN-N.

Liang *et al.* similarly identify a PTEN proteoform (PTEN-α) that initiates with the most 5′ in-frame CUG and corresponds to our PTEN-L and Hopkins *et al.* PTEN-Long. Their anti-PTEN immunoblots from cells exogenously expressing a PTEN-α construct clearly show two higher molecular weight proteins in addition to PTEN. In agreement with our study, mutation of the first in-frame CUG prevents translation of the longest protein. Furthermore, even when the first and third CUG codons (PTEN-L and PTEN-N in our study) are mutated together, an extended PTEN proteoform is still apparent which is very likely to correspond to PTEN-M. A recent report showing anti-PTEN immunoblots from matched normal and tumour tissue samples clearly identifies, in addition to canonical PTEN, at least two slower migrating endogenous PTEN proteoforms expressed in a similar ratio to PTEN-L and PTEN-M in our study [61].

When multiple proteoforms are translated from a single mRNA, the efficiency of initiation at each start codon could set the ratio of proteoform steady-state levels assuming each protein has similar stability. However, this ratio may vary during conditions in which initiation efficiency is altered. Eukaryotes have developed elaborate mechanisms for the recognition of the correct initiation codon and the levels of certain initiation factors can regulate the fidelity of initiation, especially on suboptimal (non-AUG and AUG in poor context) start codons [52,53,68]. While elevated levels of eIF1 can increase the stringency of start codon selection, elevated levels of eIF5 have the opposite effect. Here we show that overexpression of either eIF1 or eIF5 had minimal effect on the steady-state levels of PTEN proteoforms (electronic supplementary material, figure S6). This suggests that either the non-AUG initiation events in *PTEN* are refractory to normal stringency controls or the steady-state protein levels of these proteoforms are regulated tightly by rapid turnover. An alternative explanation is that because the *PTEN* 5′ leader is long, the many potential out-of-frame near-cognate codons could create uORFs and thus preclude the expected effects of eIF1/5 overexpression on translation of the PTEN N-terminal extensions.

Several groups have observed that PTEN-L can downregulate the PI3K pathway in a similar manner to PTEN [49,50,61]. *In vitro* studies comparing the catalytic activities of purified PTEN and PTEN-L reveal that both enzymes can dephosphorylate PIP3, although, interestingly, PTEN phosphatase activity can be activated by its reaction product (PIP2), whereas PTEN-L cannot and is thus constitutively active [60,67]. We tested whether the PTEN proteoforms identified in our study still retained the ability to downregulate the PI3K pathway (figure 4 and electronic supplementary material, figure S3). All PTEN proteoforms were able to reduce AKT phosphorylation to levels similar to those of canonical PTEN and PTEN-L, suggesting that the N-terminal extensions do not have major effects on the dephosphorylation activity of PTEN proteoforms.

Hopkins *et al.* [49] report that exogenously expressed PTEN-L is targeted for secretion from cells by a predicted N-terminal signal peptide and cleavage site. Furthermore, they also show that immediately following the predicted cleavage site is a functional cell re-entry signal similar to the HIV TAT protein [49]. Subsequently, Wang *et al.* [61] confirmed that PTEN-L, but not PTEN, can enter cells, although whether PTEN-L can be secreted from cells was not tested. Intriguingly, both PTEN-M and PTEN-N, while lacking the predicted signal peptide, do still retain the putative cell re-entry signal (electronic supplementary material, figure S5). We overexpressed GFP fused to both the PTEN-L (with putative secretion signal) and PTEN-M N-terminal extensions but could not detect PTEN-L-GFP in the cell culture media after concentrating by immunoprecipitation (figure 5*b*). It is not so surprising that PTEN-M-GFP is not found extracellularly because it lacks the putative signal peptide, but failure to detect PTEN-L was unexpected and suggests that most PTEN-L is not secreted. It is possible that this assay was not sensitive enough to detect low levels of secreted PTEN-L-GFP, so we further tested whether a secretion signal resided in the PTEN-L extension by fusing the *PTEN* 5′ leader to *Gaussia* luciferase. *Gaussia* luciferase is approximately 1000 times more sensitive than either *Renilla* or firefly luciferases [69], yet we could not detect any extracellular luciferase activity, above background, in constructs harbouring the putative PTEN-L signal peptide (figure 5*c*). One explanation for the discrepancy between our PTEN localization experiments and those of previous studies is that, because canonical PTEN has been found in exosomes [70,71] and can homodimerize [72], we decided to make reporters that do not contain sequences encoding the canonical *PTEN* CDS. Perhaps the important targeting signals are only ‘visible’ in the context of the full-length PTEN proteins. Yet another possible explanation for not detecting our PTEN-L chimeras in the cell culture media could be that the efficiency of cell re-entry is extremely high. However, detection of extracellular luciferase activity when the PTEN-L signal peptide is replaced with either the *Gaussia* luciferase or interleukin-2 signal peptide would argue against this possibility (figure 5*c*).

The functional significance of the PTEN-M N-terminal extension has yet to be determined but perhaps some insight may be gained from previous studies on PTEN-L. There are conflicting reports as to whether the PTEN-L extension has the potential to form a three-dimensional structure [60,67] or whether it is intrinsically disordered [65,66]. A recent elegant HDX-MS approach by Masson *et al.* [62] indicates that while most of the PTEN-L N-terminal extension is indeed intrinsically disordered, there is a potential α-helix at position 151–174 (where residue 174 is the methionine encoded by the canonical *PTEN* AUG). This peptide is protected by liposomes, suggesting an interaction with the membrane. Furthermore, this potential membrane-spanning region alters both the interfacial kinetics of PTEN-L and the protein/membrane interface, causing PTEN-L to function on membranes in a ‘scooting’ mode rather than a ‘hopping’ mode that is characteristic of PTEN [62]. All N-terminally extended proteoforms described in our study possess this potential α-helix so it will be interesting to see whether these new PTEN proteoforms act in a similar manner. It is perhaps noteworthy that we could not detect PTEN-O by immunoprecipitation with PTEN antibodies, presumably because this N-terminal extension (and not L, M and N) alters protein conformation in a manner that prevents antibody access to the PTEN antigen under native conditions. This suggests possible structural differences between the PTEN-O N-terminal extension and the other PTEN proteoforms.

In our analysis of the conserved *PTEN* uORFs, we initially hypothesized that translation of uORF1 could reduce translation of uORF2, which, because of its size (45 codons), we expect to be severely inhibitory for downstream translation. In this way, translation of uORF1 could, in theory, have an overall positive effect on main ORF translation by reducing the number of ribosomes accessing the predicted inhibitory uORF2. However, intriguingly, increasing the number of ribosomes accessing uORF2 by mutation of uORF1 appears not to be very inhibitory under the conditions tested (figure 6). Therefore, we conclude that either uORF1 is frequently passed by leaky scanning, which seems unlikely given the evidence we described previously for uORF1 translation, or ribosomes translating uORF1 can re-initiate efficiently at uORF2. Alternatively, similar results would be observed if uORF2 were not very inhibitory (i.e. permits high level re-initiation). However, when ribosomes do not translate uORF2, downstream translation increases approximately 2.5-fold at the CUG of the L-proteoform in comparison with when uORF2 is available for translation (figure 6*b*,*c*). As one would predict from the scanning model of translation initiation, removal of both uORFs further increases the level of downstream initiation a further twofold (for L-FLuc). Importantly, the presence of uORF2 affects the ratio of N-terminally extended proteoforms relative to the main ORF, but has no effect on the ratio of N-terminally extended proteoforms to each other (electronic supplementary material, figure S10*c*,*d*).

These reporter assay results raise the intriguing possibility that the deeply conserved uORFs in the *PTEN* 5′ leader may become less inhibitory for PTEN translation under, as yet unidentified, conditions that could either decrease elongation rates, which in theory would result in ribosome accumulation along uORFs and hamper scanning, or else downregulate re-initiation. There is evidence that the canonical mTOR–S6K pathway regulates re-initiation after uORFs in plants [73]. We predict that such regulation could have dramatic effects on the abundance of N-terminally extended proteoforms, especially if initiation of both uORFs were reduced.

These findings, together with the findings on PTEN-L from other groups, could have profound implications for the interpretation of previous studies on both the catalytic activity and localization of endogenous PTEN as well as the analysis of polymorphisms within the *PTEN* 5′ leader. Furthermore, the discovery of these new PTEN proteoforms could have implications for the development of PTEN-based chemotherapeutic agents.

## Supplementary Material

Merged supplemental figures (11) plus accompanying captions
