# Supplementary material for: Systematic analysis of the PTEN 5′ leader identifies a major AUU initiated proteoform
Source: Open Biol. 2016 May 25;6(5):150203. doi: 10.1098/rsob.150203 (PMC4892431; doi:10.1098/rsob.150203)

Supplementary Figure Legends

Supplementary Figure 1.
Alignment of the *PTEN* 5’ leader from the 5' most upstream in-frame stop codon (in human and mouse) to the main ORF AUG from 52 mammals. The human and mouse sequences were obtained from RefSeq. The sequences of *Callithrix jacchus* and *Papio hamadryas* were obtained using a BLAST search against the Transcriptome Shotgun Assembly (TSA) database. The other 48 sequences were obtained by a BLAST search against the Whole-Genome Shotgun contigs (wgs) database. The initial alignment was preformed with the help of the ClustalX algorithm, which was then followed by a manual re-alignment step. Insertions/deletions highlighted in red are the 3' most insertions/deletions that represent frameshifts in a given sequence. The upstream in-frame stop codon for the human sequence is the equivalent in most other organisms and is highlighted in magenta. The previously described CUG and main ORF AUG start codons are highlighted in green. Alternating red and black stripes marks codons in the previously identified N-terminal extension. Species names are on the left of each sequence. Completely conserved nucleotides are indicated by an asterisk at the bottom of the alignment. The non-AUG start sites identified in this study are underlined.

Supplementary Figure 2.
Screenshot of the 5’ end of the *PTEN* 5’ leader from GWIP-viz (hg19). RNAseq alignments are shown as green histograms and the positions of ribosome A-sites inferred from alignments of ribosome protected fragments (Riboseq) are shown as red columns. The data are aggregated from 15 ribosome profiling studies carried out in several human cultured cells. CAGE analysis from the indicated cells are from the Fantom Project [55] and are shown as black histograms. The number of reads at each nucleotide position is indicated on the y-axis. The nucleotide corresponding to the most abundant transcript site (+187 relative to RefSeq entries) is indicated with a green arrow.

Supplementary Figure 3.
Replicate immunoblots of cell lysates prepared from PTEN-null PC3 cells transfected with PTEN expressing constructs as indicated for 48 hr (serum starved for last 24 hr) and probed with antibodies against PTEN (138G6), β-actin, phospho-AKT (S473) and pan-AKT. Replicate 1 is shown in Fig. 4.

Supplementary Figure 4.
Fluorescence microscopy of live HeLa cells imaged 24 hr after transfection with the GFP constructs indicated. Scale bar is in μm.

Supplementary Figure 5.

Illustration of the PTEN protein and proteoforms PTEN-L, PTEN-M, PTEN-N and PTEN-O. The region corresponding to AUG initiated PTEN is shaded blue whereas the dashed box represents the non-AUG initiated extensions. The signal peptide and cell re-entry motif predicted by Hopkins *et al* [49] are shown as green and red boxes respectively. The putative cleavage site is depicted as a blue triangle.

Supplementary Figure 6.
Upper panel - Anti-PTEN CDS (6H2.1) immunoblot of anti-PTEN CDS (138G6) immunoprecipitates prepared from HEK-293T cells transfected with constructs overexpressing either eIF1 or eIF5. CO indicates control immunoprecipitates from cells transfected with empty vector.
Lower panel – Immunoblot of lysates prepared from cells transfected for immunoprecipitation above probed with antibodies against eIF1, eIF5 and β-actin.

Supplementary Figure 7.
Screenshot of the 5' end of the *PTEN* 5' leader from GWIP-viz (hg38) and illustration of the ORF architecture showing the two conserved AUG initiated uORFs and sites of non-AUG initiation described in this study. RNAseq alignments are shown as green histograms and the positions of ribosome A-sites inferred from alignments of ribosome protected fragments (Riboseq) are shown as red columns. The data are aggregated from 15 ribosome profiling studies carried out in several human cultured cells. The number of reads at each nucleotide position is indicated on the y-axis.

Supplementary Figure 8.
Relative luciferase activities (FLuc / RLuc) of firefly encoding sequences fused to the wild-type or mutant *PTEN* 5' leader as indicated and transfected into either MCF-7, HeLa or PC3 cells as indicated. Red crosses indicate mutation of AUG start codons to non-initiating AGG codons. L, M, N and O depict the approximate site of initiation of PTEN extensions. The dashed box represents the increase in ORF length when the stop codon of uORF1 is changed to a sense codon.

Supplementary Figure 9.
Relative luciferase activities (FLuc / RLuc) of firefly encoding sequences fused to the wild-type or 'no uAUG' mutant (constructs 1 and 5 respectively from Fig. 6) *PTEN* 5' leader as indicated and transfected into HEK-293T cells. Cells were transfected in triplicate wells (6-well plate) and transfectants processed for simultaneous RNA and protein isolation (see Materials and Methods). RNA levels were determined by RT-qPCR and relative luciferase activities were either normalised to RNA levels (red) or not (blue). Red crosses indicate mutation of AUG start codons to non-initiating AGG codons. L, M, N and O depict the approximate site of initiation of PTEN extensions.

Supplementary Figure 10.

A. Dual luciferase assay from cell lysates prepared from HEK-293T cells transfected (three biological replicates) with the indicated constructs.

B. Replicate immunoblots of cell lysates prepared from HEK-293T cells transfected with constructs 1-6 and probed with antibodies against firefly and *Renilla* luciferases. Replicate 1 is shown in Fig. 6B.

C. Within-lane densitometry analysis from three biological replicates of the proteins detected by anti-FLuc in B and Fig. 6B. Proteoforms M and N could not be resolved sufficiently from each other for accurate densitometry analysis, so the intensity of both proteins together is determined. The ratio of each proteoform is calculated as a fraction of the sum of the L, M/N and O proteoforms.

D. Within-lane densitometry analysis from three biological replicates of the proteins detected by anti-FLuc in B and Fig. 6B. The ratio of each proteoform is calculated as a fraction of the sum of L, M/N, O and main ORF proteoforms.

Supplementary Figure 11.

Relative luciferase activities (FLuc / RLuc) of firefly encoding sequences fused to the wild-type or 'no uAUG' mutant (constructs 1 and 5 respectively from Fig. 6A) *PTEN* 5' leader as indicated and transfected into HEK-293T cells for 6 hr before treatment with 5 μM arsenite for either 2 hr or 4 hr as indicated.

Supplementary Figure 1.

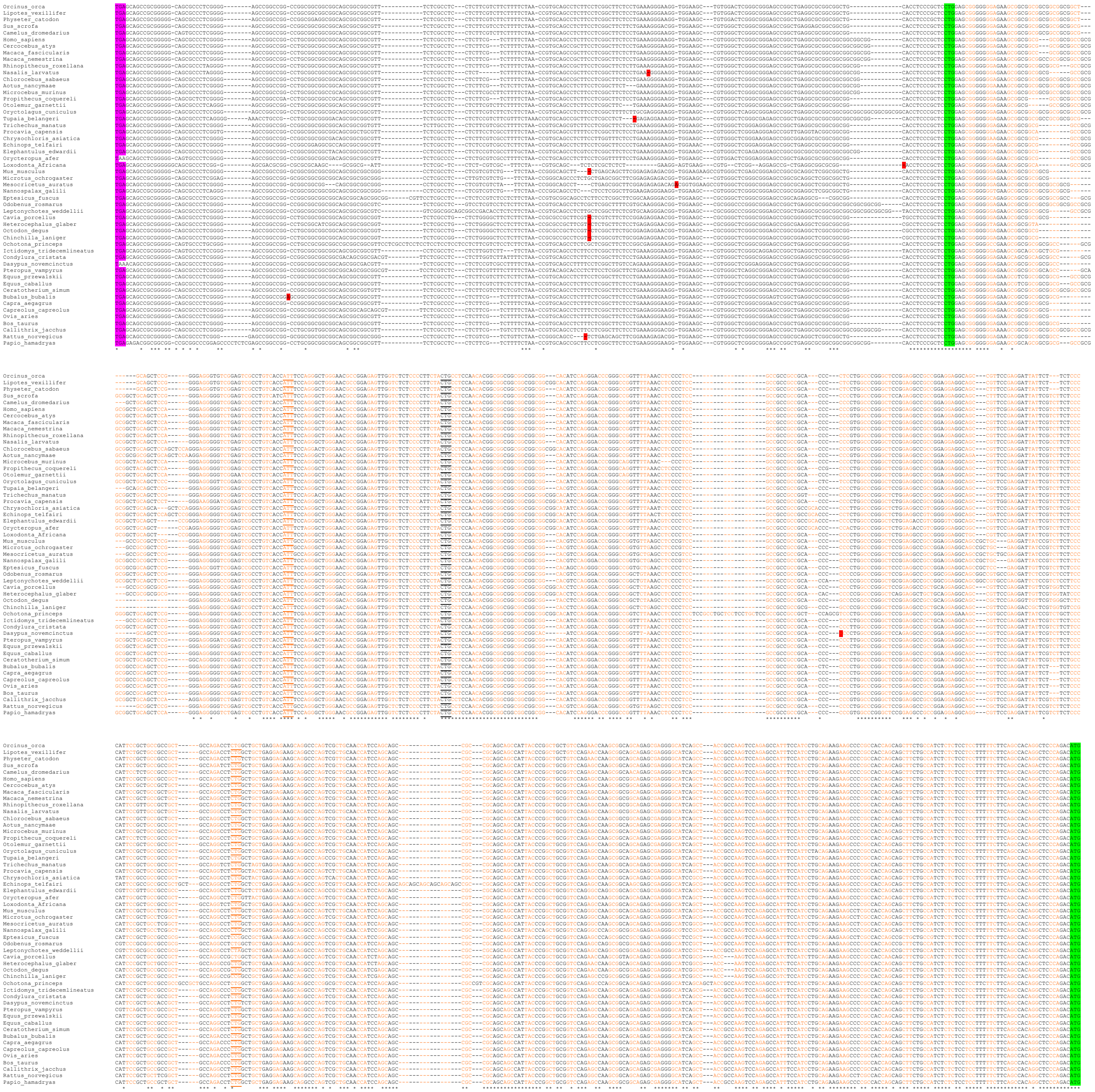

Supplementary Figure 2.

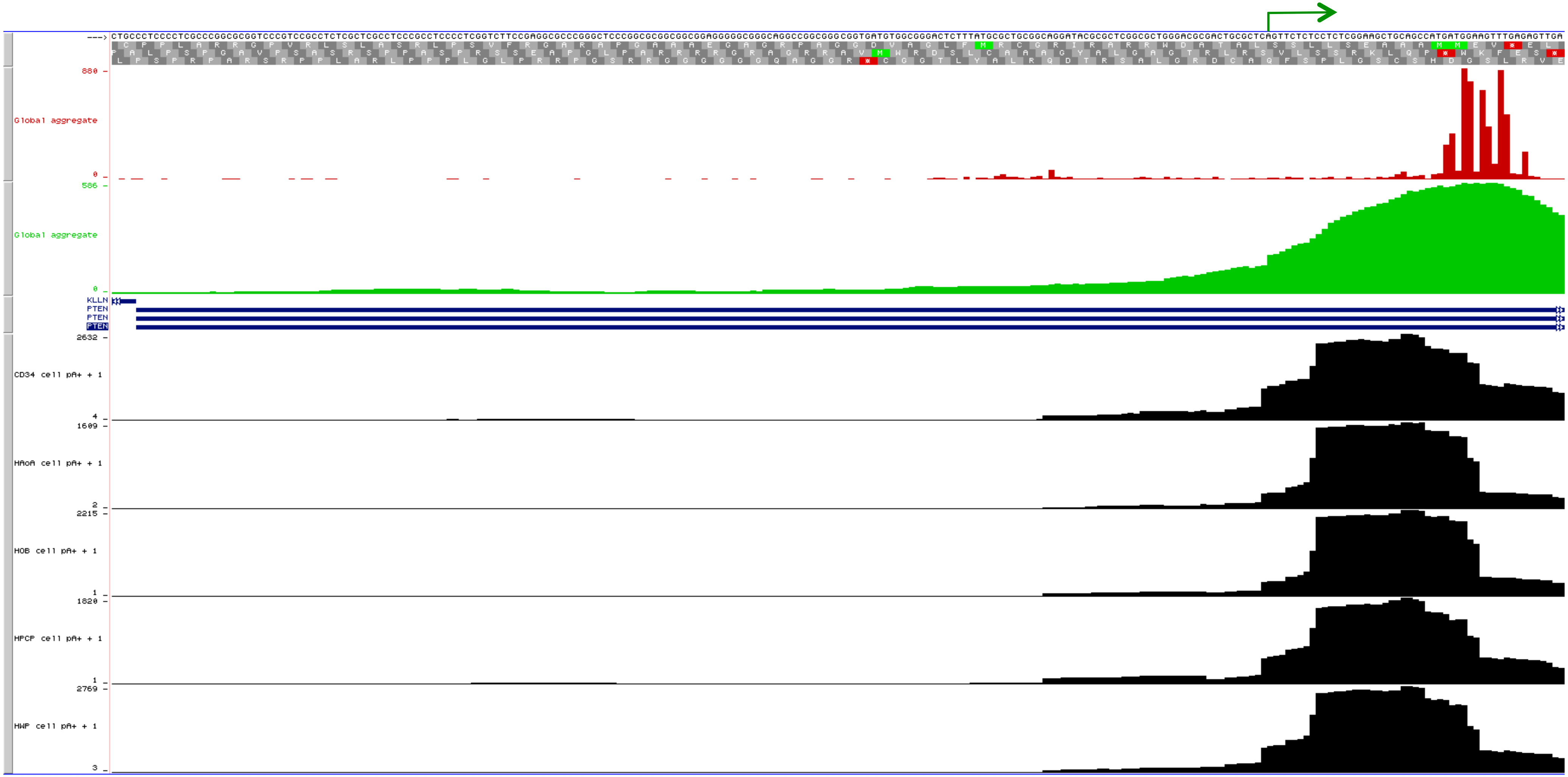

Supplementary Figure 3.

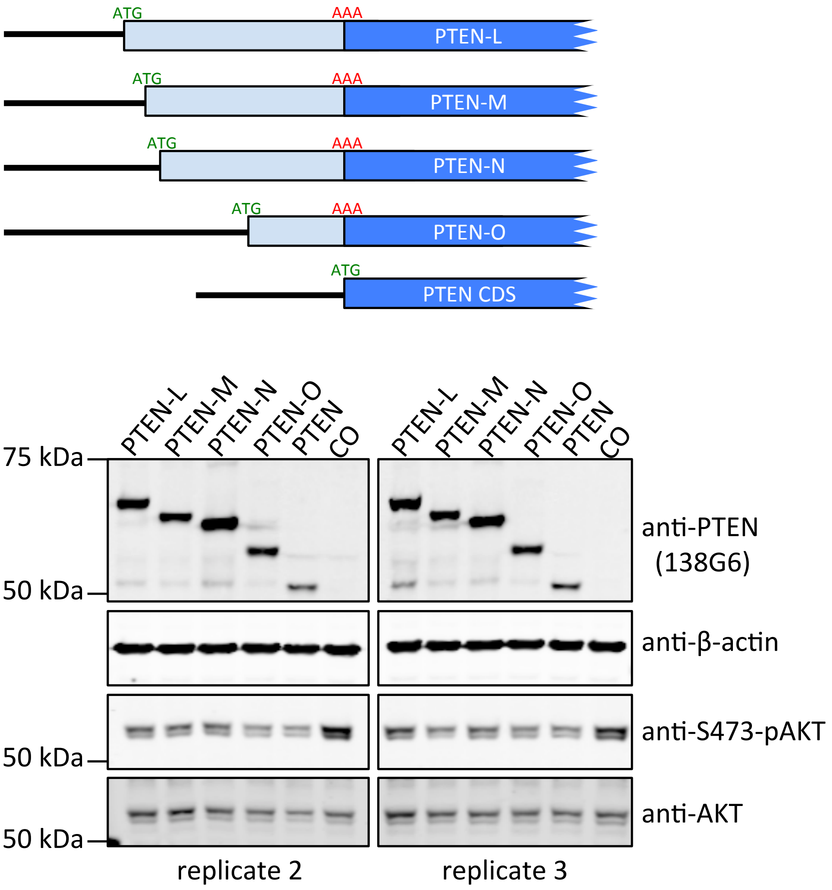

Supplementary Figure 4.

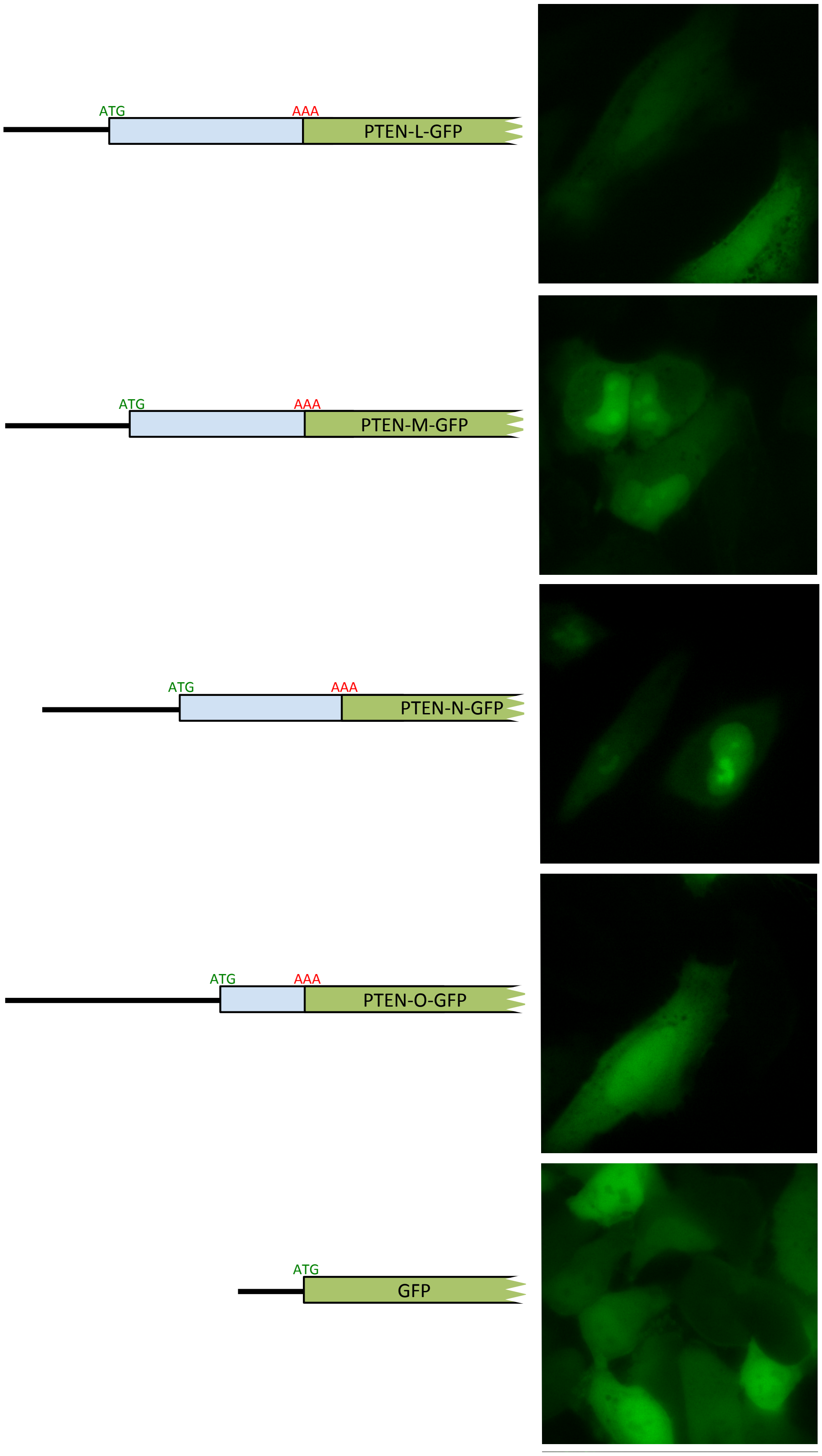

Supplementary Figure 5.

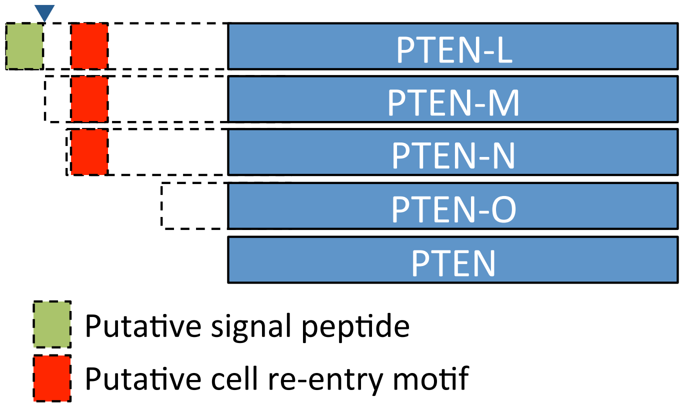

Supplementary Figure 6.

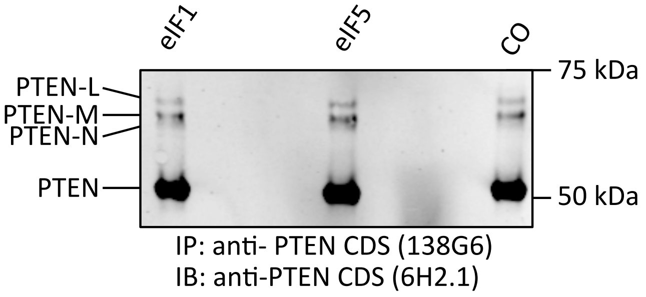

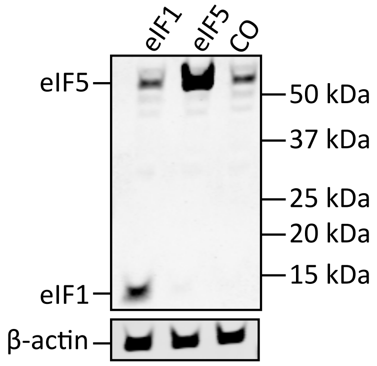

Supplementary Figure 7.

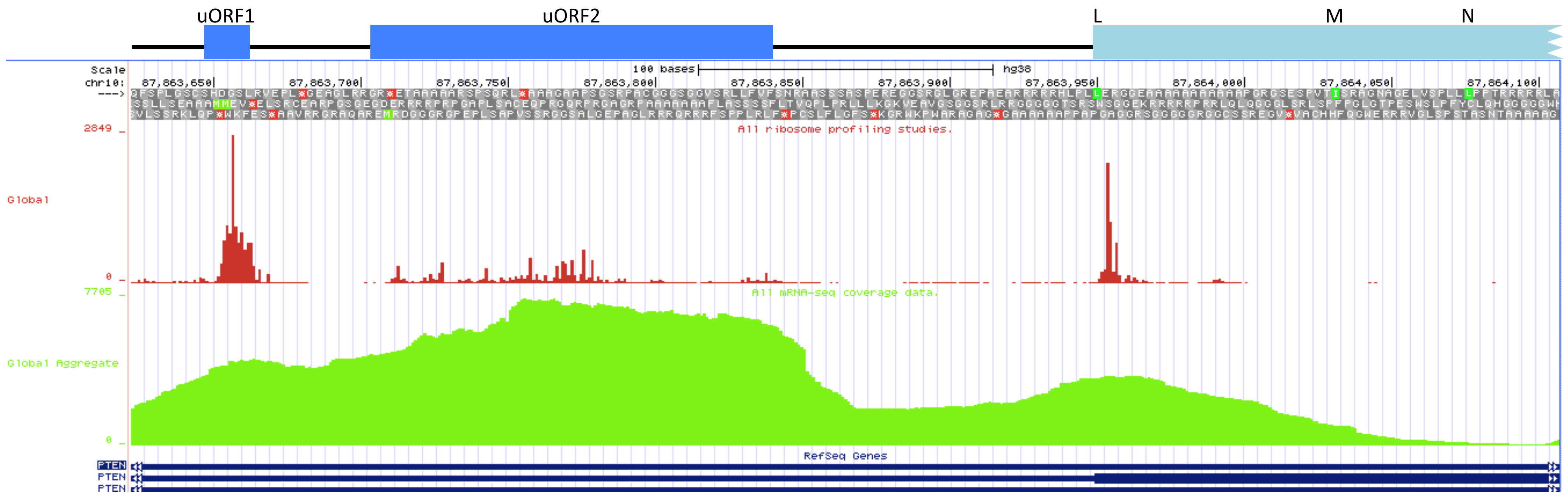

Supplementary Figure 8.

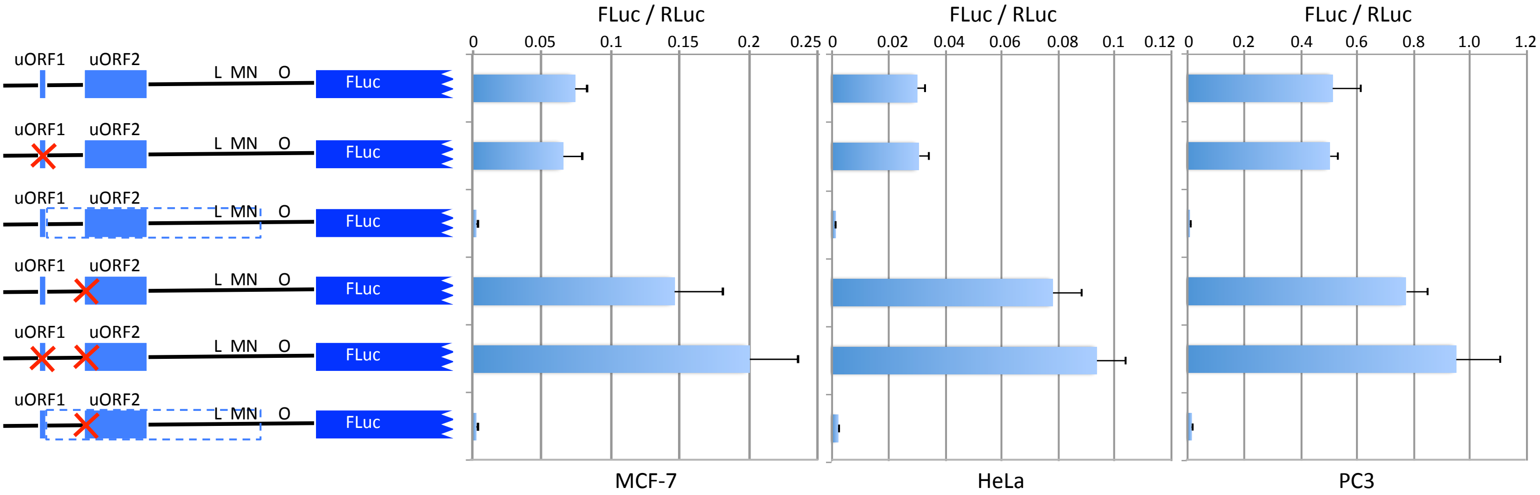

Supplementary Figure 9.

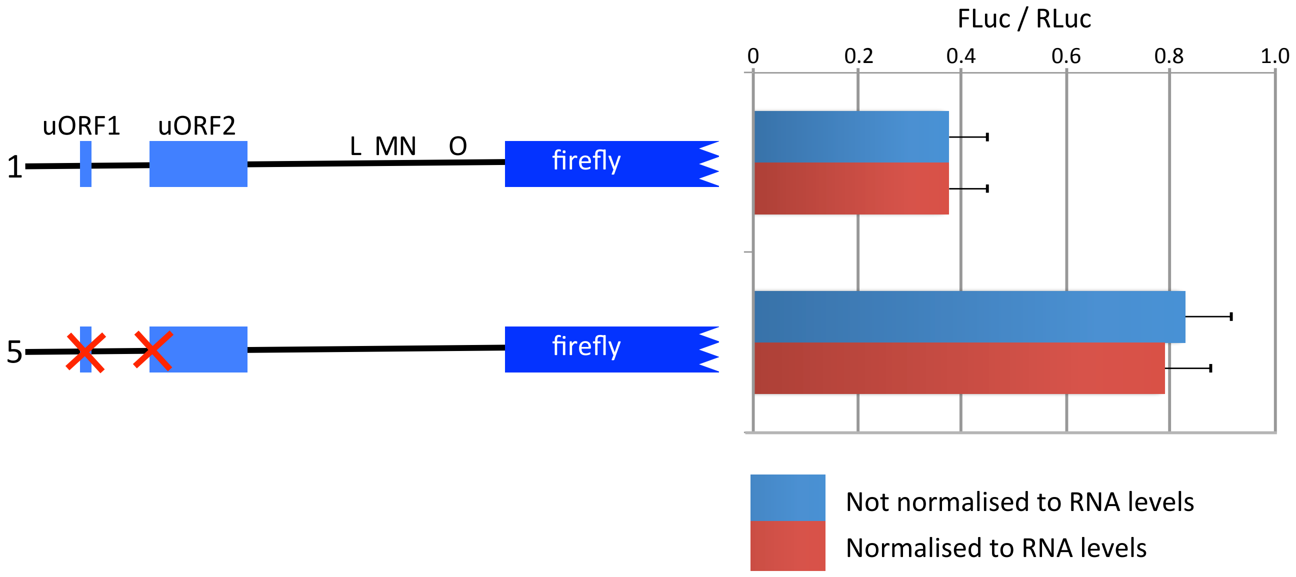

Supplementary Figure 10.

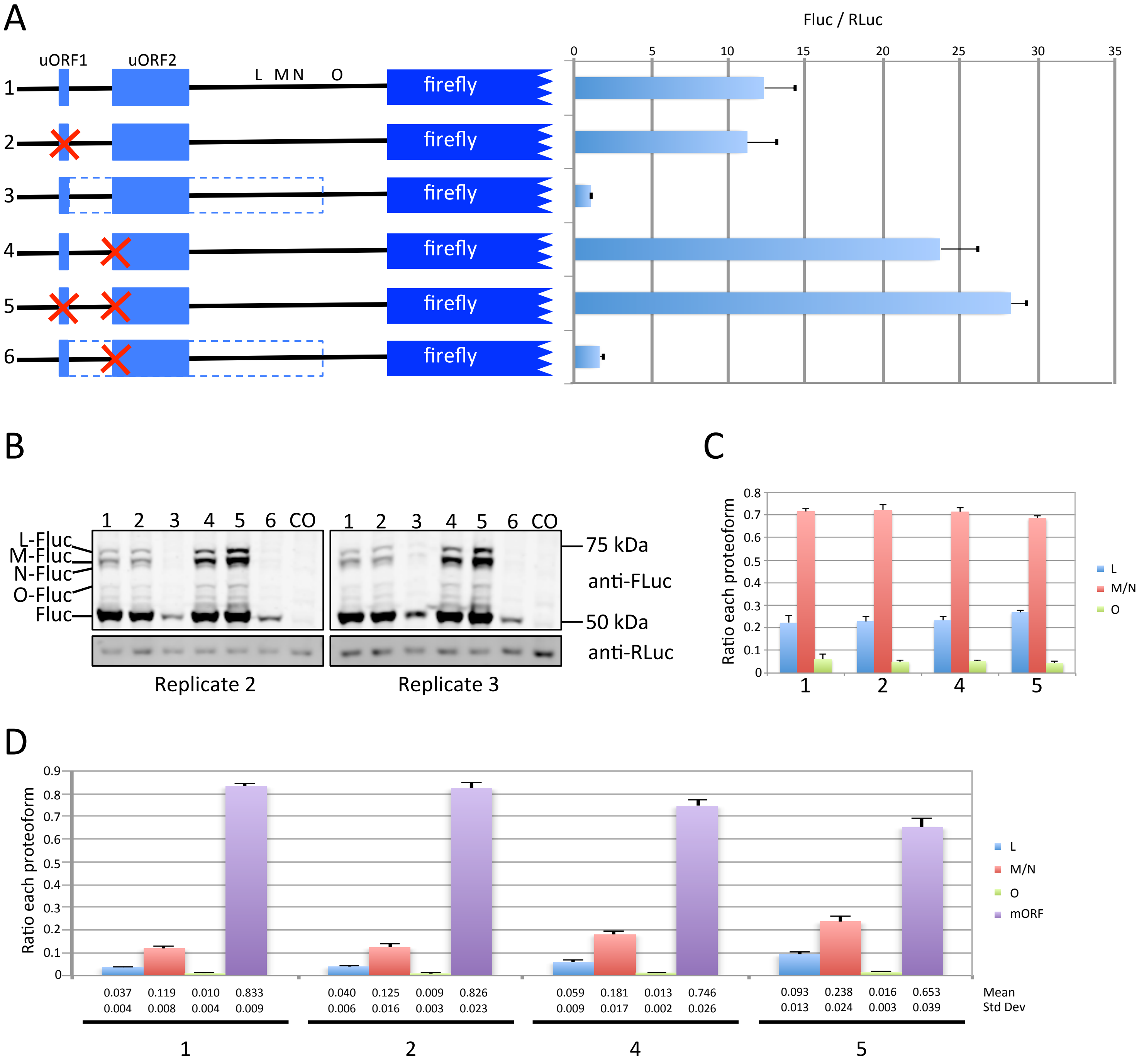

Supplementary Figure 11.

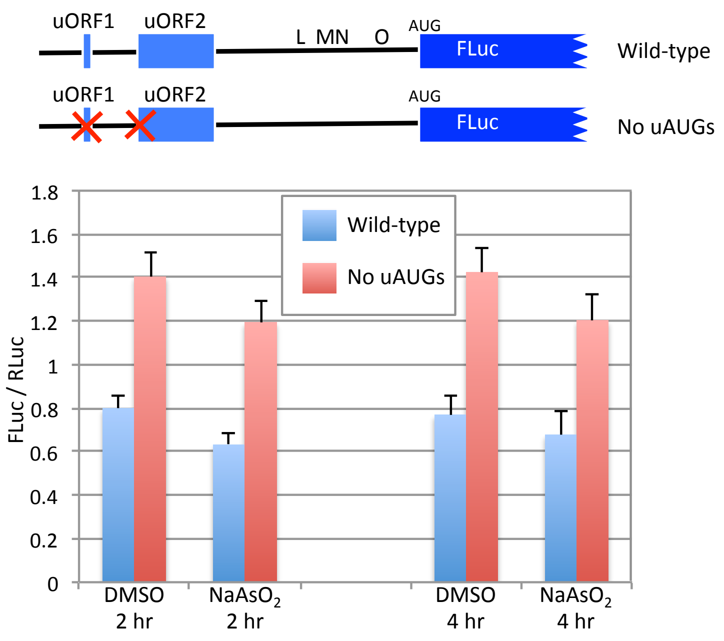

Supplement: Merged supplemental figures (11) plus accompanying captions [file rsob150203supp1.pdf]
